# USP8 protects rat-derived H9C2 cardiomyocytes from doxorubicin-triggered ferroptosis and cell death through deubiquitination-mediated stabilization of MDM4

**DOI:** 10.1186/s41065-025-00527-z

**Published:** 2025-08-14

**Authors:** Yixi Li, Xue Yang, Liang Zhang

**Affiliations:** 1 Department of Emergency Medicine, Tongde Hospital of Zhejiang Province, Hangzhou City, Zhejiang China; 2 Department of Emergency Medicine, Sir Run Run Shaw Hospital Zhejiang University School of Medicine, No.3 Qingchun East Road, Shangcheng District, Hangzhou City, Zhejiang Province China

**Keywords:** Cardiomyocytes, Doxorubicin, Ferroptosis, Deubiquitination, USP8

## Abstract

**Background:**

Acute heart failure (AHF) is a life-threatening clinical syndrome due to impaired cardiac function. Ferroptosis has emerged as a contributor to cytotoxicity in cardiomyocytes. However, the functional interplay between USP8 and ferroptosis during AHF has not been investigated.

**Methods:**

H9C2 rat cardiomyocytes were treated with doxorubicin (Dox) to establish an experimental model. Cell cytotoxicity was evaluated by measuring cell viability, LDH release, and cell death. Ferroptosis was assessed by analyzing Fe^2+^, lipid ROS, MDA, and GSH levels in treated cells. Immunoprecipitation (IP), Co-IP, and protein stabilization assays were performed to validate the USP8/murine double minute 4 (MDM4) interaction and the regulation of USP8 in MDM4. Expression of mRNA and protein was quantified by quantitative PCR and immunoblot analyses, respectively.

**Results:**

USP8 and MDM4 were downregulated in Dox-exposed H9C2 cardiomyocytes. USP8 overexpression alleviated Dox-triggered cytotoxicity and cell death in H9C2 cardiomyocytes. Moreover, USP8 overexpression mitigated H9C2 cardiomyocyte ferroptosis induced by Dox. Mechanistically, USP8 stabilized MDM4 via deubiquitination. Inhibition of MDM4 counteracted the ability of USP8 overexpression to attenuate Dox-triggered cell death and ferroptosis in H9C2 cardiomyocytes.

**Conclusion:**

Our findings indicate that USP8 overexpression protects H9C2 cardiomyocytes from Dox-induced ferroptosis by stabilizing MDM4 via deubiquitination.

**Supplementary Information:**

The online version contains supplementary material available at 10.1186/s41065-025-00527-z.

## Introduction

Acute heart failure (AHF) is a life-threatening clinical syndrome characterized by rapid onset or worsening of symptoms and hemodynamic instability due to impaired cardiac function. It remains a leading cause of global hospitalization, particularly among individuals aged over 65, with high mortality and readmission rates [1]. The etiology of AHF is multifactorial, arising from acute triggers such as myocardial infarction, myocarditis, hypertension crises, valvular dysfunction, and arrhythmias [1, 2]. Among these, cardiac ischemia-related myocardial damage is regarded as a major cause of heart failure, ultimately contributing to the impaired cardiac function [3]. Management strategies prioritize decongestion through diuretics, vasodilators, and inotropic agents to stabilize hemodynamics. Recent guidelines emphasize early initiation of guideline-directed medical therapies (GDMTs) to improve outcomes. Non-pharmacological interventions, such as mechanical ventilation or ultrafiltration, are reserved for refractory cases [4, 5]. However, current therapies primarily alleviate symptoms without addressing underlying pathophysiology. Further research is needed to elucidate molecular pathways and develop targeted therapies to address this clinical urgency.

Ferroptosis, a newly identified form of regulated cell death driven by lipid peroxidation, represents a distinct pathway differing from apoptosis or necrosis [6–8]. Recent studies implicate ferroptosis in diverse pathologies, including cancer and neurodegenerative diseases, with growing attention to its role in cardiovascular disorders [9, 10]. In myocardial tissue, ferroptosis contributes to ischemia-reperfusion injury, cardiomyopathy, and heart failure [11]. Suppression of ferroptosis has emerged as a promising intervention strategy to mitigate cardiac dysfunction [12]. Emerging evidence links ferroptosis to heart failure progression, underscoring its potential as a biomarker or intervention target.

Ubiquitination, a key protein modification in various cancer pathways, plays a pivotal role in the tumor microenvironment [13]. Ubiquitin-specific protease 8 (USP8), a deubiquitinating enzyme, regulates protein stability and cellular signaling by removing ubiquitin chains from substrates [14]. Recent research has highlighted its significant involvement in multiple pathological processes. For instance, in hepatocellular carcinoma, USP8 inhibition induces ferroptosis by destabilizing OGT, thereby disrupting SLC7A11-mediated cystine uptake [15]. In liver fibrosis, the METTL3/MALAT1/PTBP1 axis promotes macrophage pyroptosis and inflammation through USP8-mediated TAK1 regulation [16]. For Cushing’s disease, USP8 mutations predominantly occur in macroadenomas and correlate with distinct clinical phenotypes and treatment responses [17]. Intriguingly, USP8 has been shown to activate cardioprotective mitophagy in cardiomyocytes through its deubiquitinating function, thereby ameliorating myocardial injury [18]. However, the functional interplay between USP8 and ferroptosis during AHF has not been investigated.

Murine double minute 4 (MDM4), a critical regulator of the tumor suppressor p53 by modulating its ubiquitination and degradation, functions as a pro-oncogenic player in cancer [19, 20]. MDM4 interacts with the p53 signaling pathway to modulate oxidative stress and lipid peroxidation, as demonstrated in studies linking MDM4 to ferroptosis resistance in cancer models [21]. In embryonic development, MDM4 cooperates with MDM2 to control p53-dependent endocardial cushion morphogenesis, with deficiency causing congenital heart defects [22]. MDM4 maintains cardiomyocyte survival by suppressing p53-mediated apoptosis, and its loss leads to p53-dependent dilated cardiomyopathy with gender-specific progression [23].

In this study, to elucidate the role of USP8 in cardiomyocyte ferroptosis during AHF, doxorubicin (Dox)-treated H9C2 rat cardiomyocytes were employed as an experimental model. This well-established in vitro system reliably recapitulates key features of chemotherapy-induced cardiotoxicity, including oxidative stress, apoptosis, and ferroptosis [24–26]. Our findings show that USP8 overexpression exerts a protective effect against Dox-induced cytotoxicity in H9C2 cardiomyocytes by suppressing ferroptosis. Furthermore, our study demonstrates that this cytoprotective function is dependent on USP8’s deubiquitinating activity, which regulates MDM4 deubiquitination.

## Materials and methods

### Cell culture and Dox exposure

The H9C2 cardiomyocyte cell line (#SNL-029), originally isolated from rat cardiac tissues, was obtained from Sunncell (Wuhan, China). Cells were grown in DMEM (MedChemExpress, Shanghai, China) containing 10% FBS (HiMedia Laboratories, LBS Marg, Mumbai, India) and 1% antibiotic solution (penicillin-streptomycin; Beyotime, Shanghai, China). All cultures were maintained at 37 °C under a 5% CO_2_-enriched humidified environment in a Thermo incubator (Thermo Fisher Scientific, Geel, Belgium).

To assess the cardiotoxic potential of Dox, the compound was obtained from MedChemExpress. H9C2 cardiomyocytes were exposed to increasing Dox concentrations (0, 0.1, 0.5, 1, 5, and 10 µM) for different time points (24, 36, and 48 h). Cell viability was subsequently measured using the method detailed in later sections. For generating Dox-induced cardiotoxicity models in vitro, H9C2 cells were incubated with 1 µM of Dox for 24 h as described previously [27].

### Plasmid, siRNA, and transfection of cell line

pLV3-CMV-USP8(rat)-Puro (oeUSP8 denoted the USP8 overexpression plasmid), pLV3-CMV-MDM4(rat)-Puro (oeMDM4 denoted the MDM4 overexpression plasmid), and the corresponding control vector (oeNC referred to the negative control plasmid) were obtained from Miaoling Plasmid (Wuhan, China). For MDM4 depletion experiments, an Mdm4 Rat Pre-designed siRNA Set A pool (siMDM4) and a scrambled control (siNC) were used, both sourced from MedChemExpress. Transfection experiments in H9C2 cells with the applicable amount of oeNC, oeUSP8, oeUSP8 + siNC, or oeUSP8 + siMDM4 were accomplished according to the supplier’s recommended procedures (Thermo Fisher Scientific), employing Lipofectamine 3000. Following a 24-h transfection period, the engineered cells were collected and incubated with 1 µM Dox for an additional 24-h treatment.

### Cell viability assay by CCK-8 assay

In 96-well white plates (Corning, Shanghai, China), H9C2 cardiomyocytes were treated with either varying doses of Dox or transient transfection followed by Dox administration. Following treatments, each well received 10 µL of CCK-8 reagent (Biotech, Nanjing, China) and was incubated for 3 h as recommended by the supplier. Optical density measurements at 450 nm were then acquired using an Infinite M200 System (Tecan, Männedorf, Switzerland).

### Quantitative PCR

RNA preparation and gene expression analysis were carried out as follows: H9C2 cardiomyocytes, either untreated or exposed to 1 µM Dox for 24 h, were lysed for total RNA extraction using the RNeasy Mini Kit (Qiagen, Hilden, Germany). Subsequently, 500 ng of purified RNA was converted to cDNA with the iScript Kit (Bio-Rad, Glattbrugg, Switzerland). For quantitative PCR, reactions were prepared in 10 µL volumes consisting of 5 µL SYBR Green master mix (Applied Biosystems, Warrington, UK), 0.4 µL of each primer (final concentration 2.5 µM), 2 µL of cDNA template, and 2.2 µL of nuclease-free water (Servicebio, Wuhan, China). Gene expression quantification was done using the comparative Ct method (2^−ΔΔCt^), with primer sequences specific for: USP8: Forward 5’-TTTGCGTCTGAGAACGGAGG-3’, Reverse 5’-ATGAATGATTCTTGCGCAGC-3’; MDM4: Forward 5’-CAGAGGAAGGAAGGACTGTGC-3’, Reverse 5’-CACATGTATCTGTGGCGTTCG-3’. Rat β-actin served as the endogenous control, amplified using standard primer sequences (Forward 5’-CCCGCGAGTACAACCTTCTT-3’, Reverse 5’-CGCAGCGATATCGTCATCCA-3’).

### Immunoblotting

Following two washes with cold PBS, cellular proteins were extracted using RIPA buffer (Beyotime) containing protease/phosphatase inhibitor cocktail (Thermo Fisher Scientific) with periodic mixing for 30 min at 4 °C. After centrifugation (30 min, 4 °C, 14,000×g), protein quantification was performed via the BCA assay (Thermo Fisher Scientific). Equal protein quantities (20–30 µg) were separated electrophoretically using 4–12% Bis-Tris Plus Gel (Invitrogen, Wesel, Germany). The resulting gels were blotted to nitrocellulose membranes (Millpore, Molsheim, France) and subjected to overnight incubation at 4 °C with primary antibodies recognizing USP8 (#sc-365481, mouse monoclonal, 1:800, Santa Cruz Biotechnology, Santa Cruz, CA, USA), MDM4 (#sc-374147, mouse monoclonal, 1:5,000, Santa Cruz Biotechnology), GPX4 (#ab252833, rat monoclonal, 1:1,000, Abcam, Cambridge, UK), SLC7A11 (#ab307601, rabbit monoclonal, 1:1,000, Abcam), ubiquitin (Ub, #ab134953, rabbit monoclonal, 1:6,000, Abcam), and β-actin (#ab8226, mouse monoclonal, 1:1,000, Abcam). Following incubation with secondary antibodies labeled with HRP, the ECL Detection reagent (GE Healthcare, Little Chalfont, UK) was used for chemiluminescent imaging using Fluorochem M imaging system (Protein Simple, San Jose, CA, USA).

### LDH release measurement

To assess cellular damage, LDH release was measured in H9C2 cardiomyocytes following either Dox treatment (1 µM, 24 h) or transfection prior to Dox exposure, using a commercial LDH Release Assay Kit as suggested by the vendor (Beyotime). Absorbance readings at 450 nm wavelength were obtained with an Infinite M200 microplate reader.

### Cell death assay by flow cytometry

Apoptotic evaluation was performed by dual-labeling with FITC-conjugated Annexin V and propidium iodide (BD Biosciences, Heidelberg, Germany) in H9C2 cardiomyocytes subjected to Dox treatment (1 µM, 24 h) or transfection before Dox exposure. Cell populations were analyzed by flow cytometry (Epics XL-MCL, Beckman Coulter, Fullerton, CA, USA).

### Detection of Fe^2+^, lipid ROS, MDA, and GSH levels

Intracellular Fe^2+^, MDA, and GSH levels in H9C2 cardiomyocytes subjected to Dox treatment (1 µM, 24 h) or transfection before Dox exposure were quantified. For these measurements, standardized commercial kits were utilized, including the Ferrous Iron Assay Kit (Elabscience, Wuhan, China), GSH Assay Kit (Elabscience), and MDA Assay Kit (Spbio), as per the manufactory protocols. The Tecan Infinite M200 system was used to measure absorbance values. The BODIPY-C11 fluorescent probe (5 µM; Thermo Fisher Scientific) was used to quantify lipid ROS by measuring fluorescence shifts after 30 min incubation at 37 °C. Following PBS washes, samples were analyzed using an Epics XL-MCL flow cytometer per standard protocols.

### Immunoprecipitation (IP) and Co-IP experiments

To perform IP assays, a commercial IP Assay Kit was used following the accompanying guidelines (Beyotime). Total protein lysates were prepared from H9C2 cardiomyocytes either treated with 1 µM Dox for 24 h or transfected prior to Dox exposure. These lysates were subsequently incubated with Protein A magnetic beads that had been pre-coated with antibody against MDM4 (#MA5-16112, Thermo Fisher Scientific) or IgG (#B900620, Proteintech, Wuhan, China). Following overnight incubation at 4 °C with gentle rotation, the immunocomplexes were collected. The captured proteins were released through thermal denaturation (95 °C, 5 min) in SDS sample buffer, enabling subsequent immunoblot detection of USP8, MDM4, and ubiquitinated MDM4 levels.

### Protein stabilization assay

To assess USP8’s impact on MDM4 protein stability, H9C2 cardiomyocytes transfected with oeNC or oeUSP8 were maintained in complete medium supplemented with 20 ng/mL of cycloheximide (CHX, InvivoChem, Guangzhou, China) for specified durations (0, 6, and 9 h). Immunoblotting with the specific antibody to MDM4 was used to quantify the levels of the remaining MDM4 protein.

### Statistics

All results were expressed as mean values with standard deviations (three biological replicates). Statistical significance (*P* < 0.05) was determined by either unpaired Student’s *t*-test or ANOVA with appropriate post-hoc comparisons (Tukey’s or Sidak’s tests), unless noted otherwise.

## Results

### USP8 and MDM4 are downregulated in Dox-exposed H9C2 cardiomyocytes

**Fig. 1:**
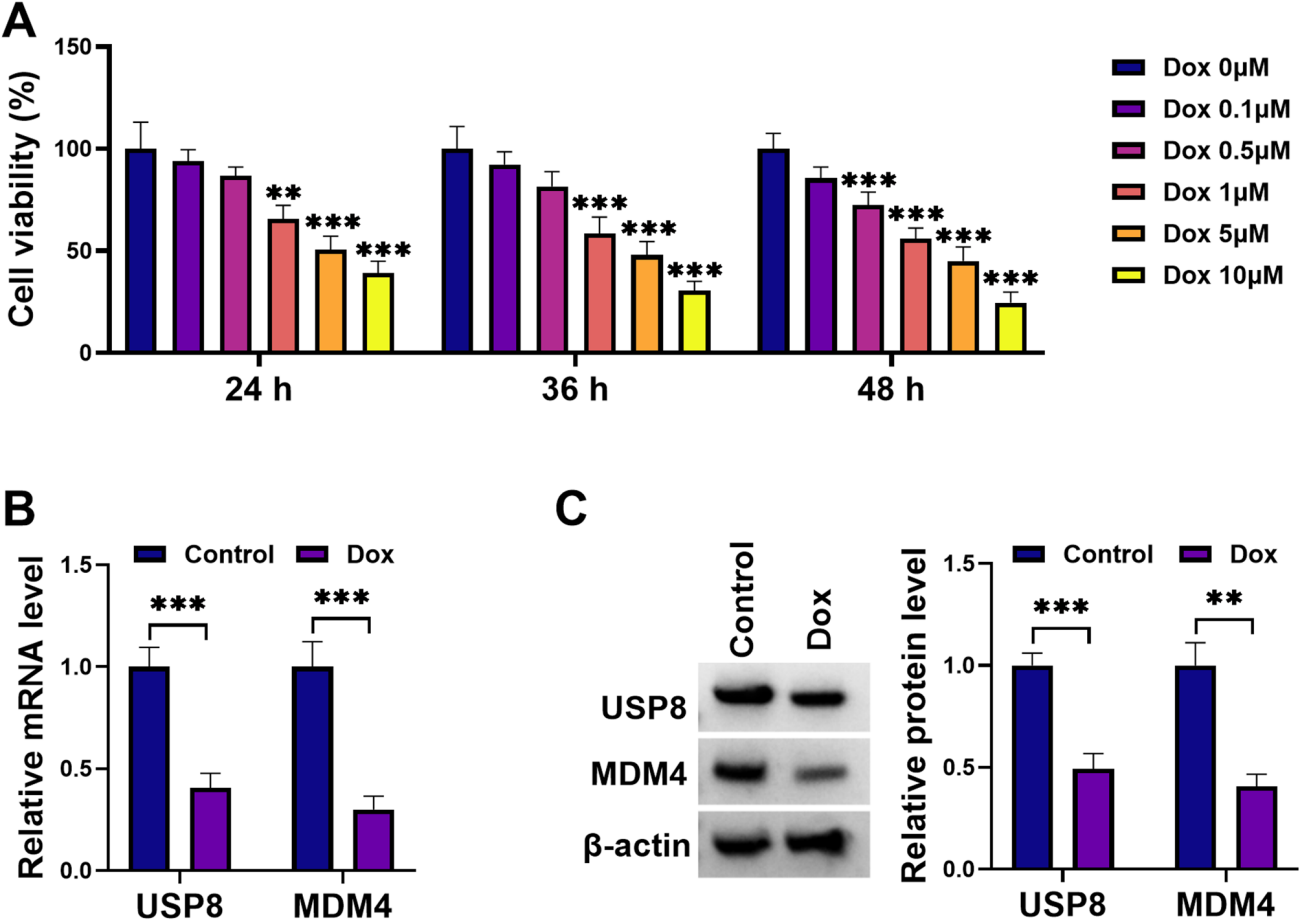
Dox exposure decreases the expression of USP8 and MDM4 in H9C2 cardiomyocytes. (**A**) H9C2 cardiomyocytes were exposed to various concentrations of Dox (0, 0.1, 0.5, 1, 5, and 10 µM) for different time frames (24, 36, and 48 h), and cells were then subjected to viability analysis by CCK-8 assay. (**B** and **C**) H9C2 cells were stimulated with or without 1 µM Dox for 24 h, and total RNA was prepared for quantitative PCR of USP8 and MDM4 mRNA transcripts (**B**), and cell extracts were collected for immunoblot analysis of USP8 and MDM4 protein levels (**C**). **P < 0.01, ***P < 0.001

Dox-induced cellular models are pivotal in studying AHF mechanisms due to drug-caused cardiotoxicity [24, 26]. To establish in vitro models of Dox-induced cardiotoxicity, H9C2 cardiomyocytes were exposed to various concentrations of Dox (0–10 µM) for different time frames (24, 36, and 48 h). As observed in Fig. 1A, Dox exposure led to a reduction in cell viability. For the induction of Dox-induced cardiotoxicity in vitro, H9C2 cells were exposed to 1 µM Dox for 24 h, following established protocols [27].

USP8 has been shown to enhance cardioprotective mitophagy in cardiomyocytes by its deubiquitinating activity, protecting against myocardial injury [18]. Thus, this study elucidated the role of USP8 in Dox-triggered cell death and ferroptosis in H9C2 cardiomyocytes. Our quantitative PCR and immunoblot analyses showed that Dox exposure significantly decreased USP8 expression at both mRNA and protein levels in H9C2 cells compared to control cells (Fig. 1B and C).

Earlier work highlights that MDM4 inhibits p53 activity in cardiomyocytes, essential for preventing p53-mediated apoptosis and maintaining heart function [23]. MDM4 is also found to be correlated with ferroptosis [21]. Intriguingly, our search for candidate substrates of USP8 using the UbiBrowser^2.0^ bioinformatics tool revealed a potential interaction between USP8 and MDM4 (Supplementary Fig. 1). Furthermore, treatment of Dox led to a striking reduction in MDM4 mRNA and protein levels compared with those in control cells (Fig. 1B and C).

### USP8 overexpression alleviates Dox-triggered cytotoxicity in H9C2 cardiomyocytes

**Fig. 2:**
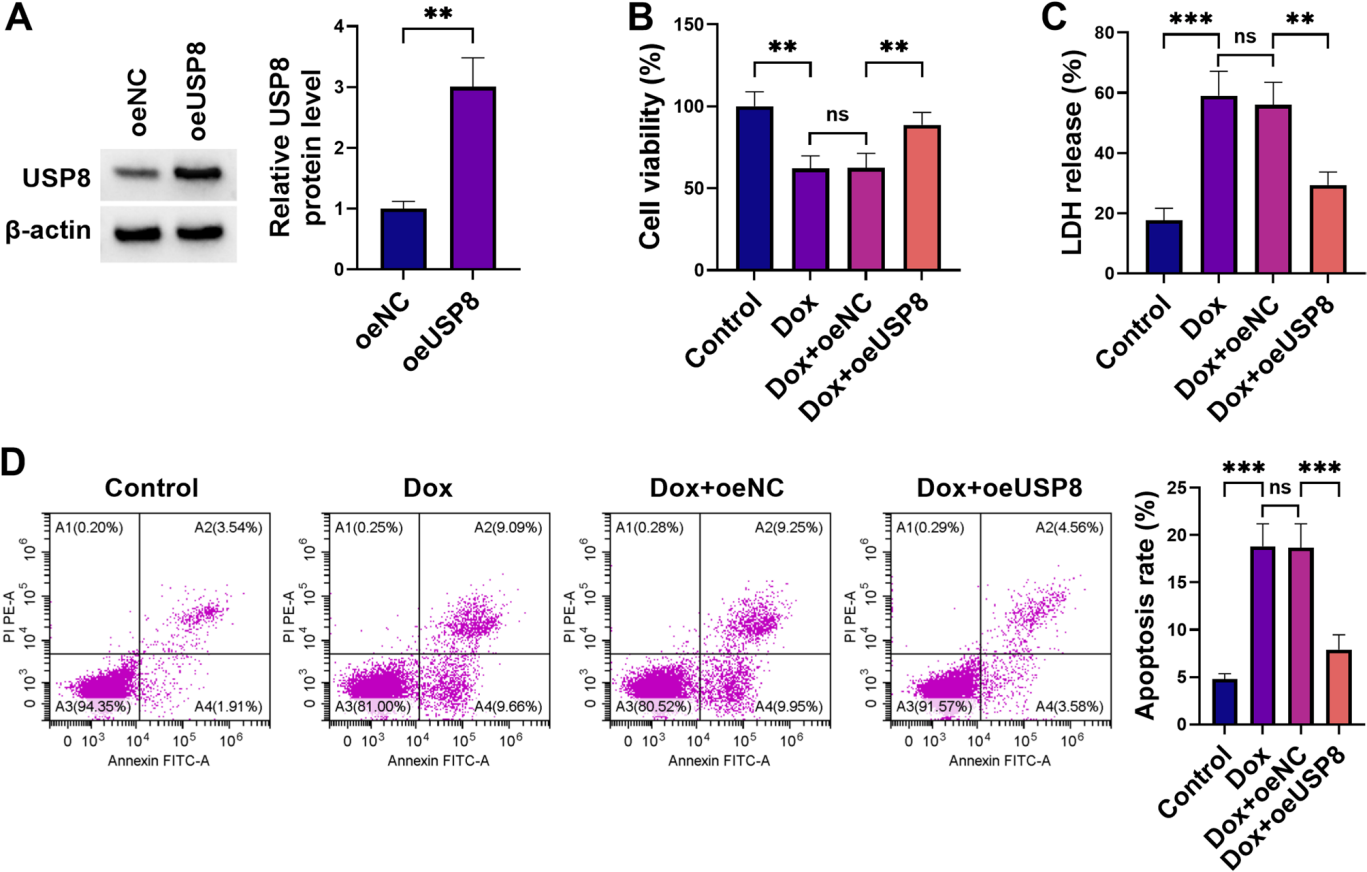
Elevated USP8 expression protects H9C2 cardiomyocytes from Dox-mediated cytotoxicity and cell death. (**A**) H9C2 cardiomyocytes were subjected to transfection with oeUSP8 or oeNC before Dox exposure, and cell extracts were collected for immunoblot analysis of USP8 protein, with β-actin as a loading control. (**B-D**) H9C2 cardiomyocytes after 24 h transfection with or without oeUSP8 or oeNC were stimulated with or without 1 µM Dox for 24 h. (**B**) CCK-8 reagent was added to the cells, and absorbance was measured. Viability was determined relative to control cells. (**C**) Cells were harvested for evaluation of LDH release content using the assay kit. (**D**) Cells were collected for flow cytometry with Annexin V/PI staining, and cell death rate per group was quantified. **P < 0.01, ***P < 0.001, ns: non-significant

Our study then studied the impact of USP8 on H9C2 cytotoxicity induced by Dox. To this end, USP8 expression was increased in Dox-exposed H9C2 cells because its downregulation upon Dox stimulation. Immunoblot analysis confirmed that introduction of a USP8 DNA construct (oeUSP8), but not the oeNC control expressing a scrambled sequence, strikingly increased USP8 protein levels in Dox-exposed H9C2 cells (Fig. 2A). Additionally, compared with the control and oeNC groups, oeUSP8 transfection increased USP8 protein levels in untreated H9C2 cells (Supplementary Fig. 2). In contrast, Dox exposure caused viability impairment in H9C2 cells, which could be rescued by increased USP8 levels (Fig. 2B). High LDH levels are linked to cell injuries during AHF processes [28, 29]. Dox treatment resulted in enhanced LDH release in the cardiomyocyte line, while overexpression of USP8 strongly abolished this effect (Fig. 2C). Consistently, Dox exposure triggered cell death in H9C2 cardiomyocytes, which could be markedly attenuated by elevated expression of USP8 (Fig. 2D). All these data suggest that USP8 plays a critical role against Dox-mediated cytotoxicity in H9C2 cardiomyocytes.

### USP8 overexpression mitigates H9C2 cardiomyocyte ferroptosis induced by Dox

**Fig. 3:**
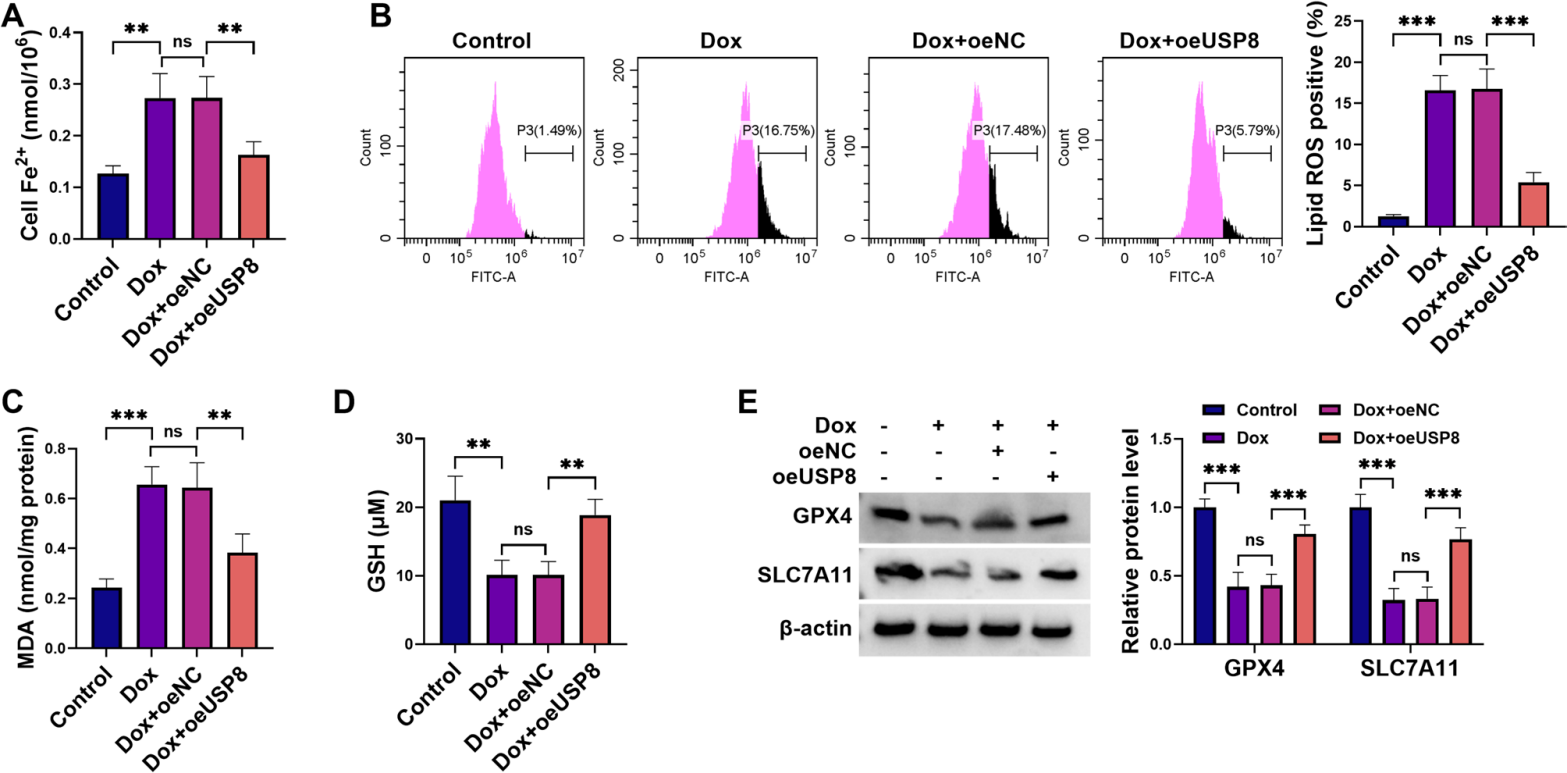
Overexpression of USP8 mitigates Dox-triggered ferroptosis in H9C2 cardiomyocytes. (**A-E**) After 24 h of transfection (oeUSP8, oeNC, or untransfected controls), H9C2 cardiomyocytes were exposed to 1 µM Dox or maintained in control medium for 24 h. (**A**) Cells were harvested for the assessment of intracellular Fe^2+^ levels using the assay kit. (**B**) Cells were subjected to BODIPY-C11 probe staining and flow cytometry for the analysis of lipid ROS production. (**C** and **D**) Cells were processed for MDA and GSH expression quantification using the commercial kits. (**E**) Cell extracts were collected for immunoblot analysis of GPX4 and SLC7A11, with β-actin as a loading control. **P < 0.01, ***P < 0.001, ns: non-significant

Next, our study investigated its potential involvement in Dox-triggered ferroptosis in H9C2 cardiomyocytes. Dox stimulation triggered characteristic ferroptotic changes including Fe^2+^ overload, increased lipid ROS levels, elevated MDA amount, and antioxidant depletion (reduced GSH), all of which were counteracted by overexpression of USP8 (Fig. 3A and D). Our immunoblot assays revealed that Dox exposure markedly decreased protein levels of the ferroptosis inhibitors GPX4 and SLC7A11 in H9C2 cardiomyocytes, while increased expression of USP8 effectively restored their levels (Fig. 3E). Collectively, our data establish that USP8 overexpression confers protection against Dox-induced cytotoxicity by suppressing ferroptosis in H9C2 cardiomyocytes.

### USP8 stabilizes MDM4 protein via deubiquitination

**Fig. 4:**
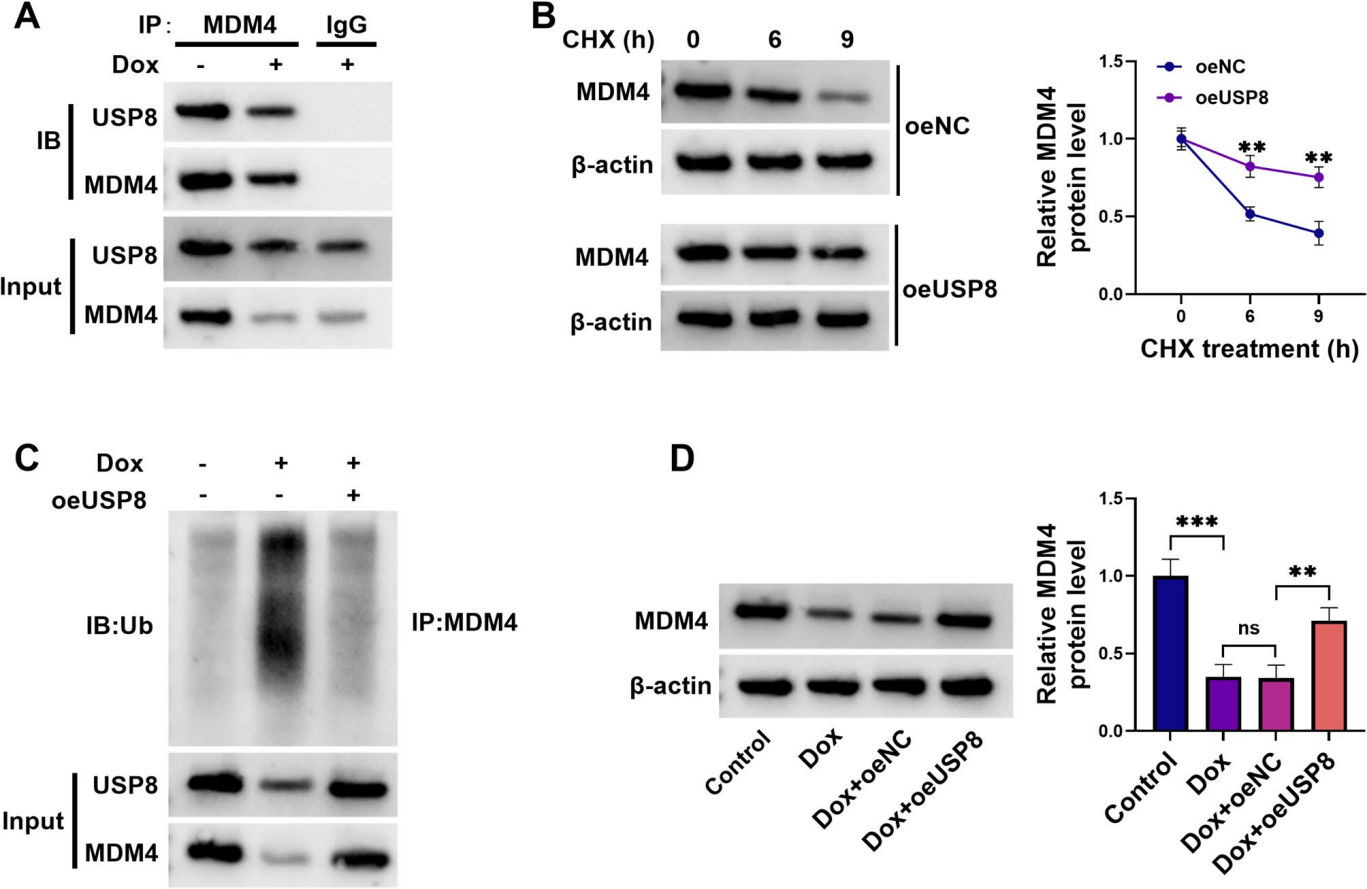
USP8 mediates MDM4 protein stabilization through its deubiquitinase activity. (**A**) H9C2 cardiomyocytes were exposed to 1 µM Dox or maintained in control medium for 24 h, and cell extracts were harvested for Co-IP assays using anti-MDM4 or anti-IgG antibody. The immunoprecipitates were subjected to immunoblotting (IB) of USP8 and MDM4. (**B**) H9C2 cardiomyocytes transfected with oeNC or oeUSP8 were treated with CHX for 0, 6, and 9 h, and cell extracts were harvested for immunblot analysis of MDM4 and β-actin. (**C**) Untransfected or oeUSP8-transfected H9C2 cardiomyocytes were treated with or without 1 µM Dox for 24 h, and cell extracts were harvested for Co-IP assays using anti-MDM4 antibody. The immunoprecipitates were processed for immunoblotting (IB) of ubiquitin-modified MDM4. (**D**) H9C2 cardiomyocytes with or without transfection were exposed to 1 µM Dox or maintained in control medium for 24 h, and cell extracts were harvested for immunblot analysis of MDM4 protein. **P < 0.01, ***P < 0.001, ns: non-significant

The UbiBrowser^2.0^ algorithm predicted that MDM4 was a candidate substrate of USP8 (Supplementary Fig. 1), with a confidence score of 0.826, inferred DUB recognition motif score of 48.9624, the motif enriched GO term pair: GO:190,090-GO:0042177, and supporting evidence including a Domain_likelihood ratio of 4.24, Go_likelihood ratio of 1.99, Network_likelihood ratio of 1, and Motif_likelihood ratio of 4.28. The algorithm also predicted that the most enriched domain pair: USP8-PF00443: ubiquitin carboxyl-terminal hydrolase–MDM4-PF02201: SWIB/MDM2 domain, with domain enrichment ratio of 8.86. To delineate the functional role of USP8 in MDM4 regulation, this study first elucidated the physical interaction between these two proteins. Co-immunoprecipitation (Co-IP) assays performed with an MDM4-specific antibody followed by immunoblot analysis demonstrated that USP8 was detected in MDM4-associated immunoprecipitates (Fig. 4A). To evaluate the functional consequence of this interaction, our study then investigated whether USP8 expression modulates MDM4 protein stabilization and expression levels. In the presence of CHX to block new protein synthesis, USP8 overexpression increased the levels of the residual MDM4 protein (Fig. 4B), indicating that USP8 was able to stabilize MDM4 protein. Although Dox reduced MDM4 protein levels in H9C2 cardiomyocytes, it indeed increased the levels of ubiquitinated MDM4 (Fig. 4C). Notably, overexpression of USP8 strongly attenuated MDM4 ubiquitination and degradation in Dox-exposed H9C2 cardiomyocytes (Fig. 4C). Moreover, increased USP8 expression significantly upregulated the levels of MDM4 protein in H9C2 cardiomyocytes stimulated with Dox (Fig. 4D). Together, these findings establish that USP8 deubiquitinates and stabilizes MDM4.

### Inhibition of MDM4 counteracts the ability of USP8 overexpression to attenuate Dox-triggered cell death and ferroptosis in H9C2 cardiomyocytes

**Fig. 5:**
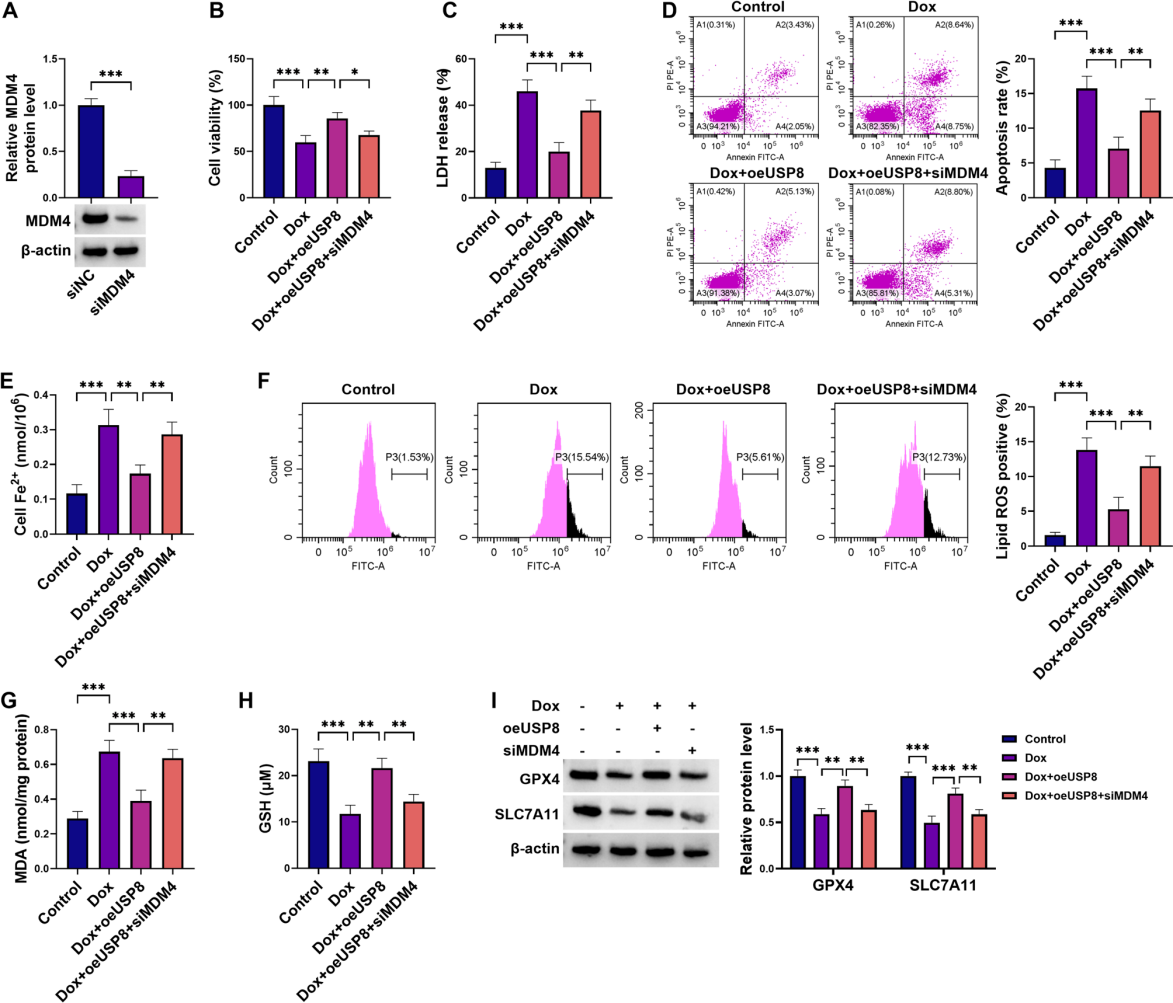
MDM4 inhibition reverses the cardioprotective effects of USP8 upregulation against Dox-triggered cell death and ferroptosis in H9C2 cells. (**A**) H9C2 cardiomyocytes were subjected to transfection with siMDM4 or siNC, and cell extracts were collected for immunoblot analysis of MDM4 protein. (**B-I**) H9C2 cardiomyocytes after 24 h transfection with or without oeUSP8 + siNC or oeUSP8 + siMDM4 were stimulated with or without 1 µM Dox for 24 h. (**B**) CCK-8 reagent was added the cells, and absorbance was measured. Viability was determined relative to control cells. (**C**) LDH release was measured in cell supernatants using the assay kit. (**D**) Cells were harvested for flow cytometry after Annexin V/PI staining, and cell death rate per group was quantified. (**E**) Intracellular Fe^2+^ levels were determined using a commercial iron assay kit. (**F**) Cells were subjected to BODIPY-C11 probe staining and flow cytometry for the analysis of lipid ROS production. (**G** and **H**) Cells were processed for MDA and GSH expression quantification using the commercial kits. (**I**) Cell extracts were prepared for immunoblot analysis of GPX4 and SLC7A11. *P < 0.05, **P < 0.01, ***P < 0.001

Having established USP8’s regulation in MDM4, our study investigated whether MDM4 functionally contributes to USP8-dependent modulation of Dox-induced cytotoxicity. To address this, MDM4 loss-of-function experiments were performed in USP8-overexpressed H9C2 cells under Dox exposure. A MDM4-specific siRNA (siMDM4) was used to silence MDM4 and its depletion efficiency was confirmed by immunoblot analysis (Fig. 5A). Transfection of oeUSP8 increased USP8 and MDM4 protein levels in Dox-treated H9C2 cells (Supplementary Fig. 3). Notably, siMDM4 transfection effectively knocked down MDM4 expression without altering USP8 levels in USP8-overexpressed H9C2 cells under Dox exposure (Supplementary Fig. 3). As expected, MDM4 loss-of-function had a counteracting impact on USP8 overexpression-driven viability enhancement (Fig. 5B), LDH release reduction (Fig. 5C), and cell death repression (Fig. 5D) in Dox-stimulated H9C2 cardiomyocytes. Furthermore, MDM4 loss-of-function remarkably reversed the protective effects of USP8 overexpression, abrogating its suppression of Fe^2+^ accumulation, lipid ROS, and MDA levels (Fig. 5E and G), while restoring GSH production, GPX4 expression, and SLC7A11 levels (Fig. 5H and I) in H9C2 cardiomyocytes under Dox exposure. In addition, oeMDM4-mediated MDM4 upregulation significantly enhanced cell viability, reduced LDH release, suppressed cell death, decreased Fe^2+^, lipid ROS, and MDA levels, increased GSH content, as well as elevated GPX4 and SLC7A11 levels in Dox-stimulated H9C2 cells (Supplementary Fig. 4). Collectively, our data demonstrate the functional involvement of MDM4 in USP8-mediated H9C2 cytoprotection during Dox exposure.

## Discussion

Dox induces cardiotoxicity through multifaceted mechanisms including oxidative stress, mitochondrial damage, and activation of regulatory cell death pathways, making it a robust model for studying AHF [26, 30]. Recent studies have established that Dox induces lipid peroxidation and cardiomyocyte ferroptosis, thereby contributing to cardiomyocyte loss and heart failure [24, 25]. Our experimental data corroborate these findings, demonstrating that Dox treatment in H9C2 cardiomyocytes significantly reduces cell viability while concurrently activating cell death and ferroptosis pathways. The observed ferroptosis induction further highlights the utility of this model for investigating iron-dependent lipid peroxidation mechanisms in cardiac pathology.

Emerging evidence positions USP8 as a critical regulator of ferroptosis, primarily through its deubiquitinating activity. For example, USP8 stabilizes GPX4 via deubiquitination, protecting intestinal cells from ferroptosis [31]. In hepatocellular carcinoma models, USP8 inhibition suppresses tumor growth and metastasis by inducing ferroptosis through OGT destabilization to disrupt OGT-SLC7A11 axis [15]; USP8 promotes cancer progression by stabilizing β-catenin and inhibiting ferroptosis [32]. These findings suggest a conserved role for USP8 in maintaining redox homeostasis across tissues. In diabetic mouse models, USP8 expression is downregulated, leading to impaired mitophagy and cardiac dysfunction. USP8 is able to enhance cardioprotective mitophagy in cardiomyocytes by removing K6-linked ubiquitin chains from parkin, thereby mitigating myocardial damage [18]. In this study, our results show that Dox downregulates USP8 expression in H9C2 cardiomyocytes. Crucially, USP8 overexpression confers protection against Dox-induced cytotoxicity by preserving cell viability and reducing apoptotic and ferroptotic signatures. This study unveils a novel USP8-dependent axis that counteracts Dox-driven ferroptosis in cardiomyocytes, positioning USP8 as a potential therapeutic target for AHF.

Emerging evidence highlights the regulatory role of MDM4 in cellular stress responses and ferroptosis regulation. For example, in colorectal cancer, MDM4 stabilizes GPX4 via TRIM21-mediated ubiquitination switching to repress ferroptosis, enhancing chemoresistance and tumor progression in p53-mutant contexts [21]. Conversely, MDM4 enhances ferroptosis by modulating lipid metabolism via PPARα activation and suppressing FSP1/CoQ10 antioxidant defense [33]. These findings suggest that MDM4 exhibits context-dependent dual roles in ferroptosis regulation. Beyond cancer, MDM4 has been implicated in other pathological states. For instance, in pulmonary fibrosis, MDM4 promotes fibrogenesis, and its inhibition reduces fibrosis by activating p53 [34]. In rheumatoid arthritis, MDM4 polymorphisms (e.g., rs4245739) show no direct RA association, but MDM2-MDM4-p53 interactions may modulate autoimmune risk [35]. In CNS injury, MDM4 deletion enhances axonal regeneration via IGF1R, suggesting therapeutic potential for neurological recovery [36]. Interestingly, MDM4 is found to participate in various cardiovascular diseases, where its dysregulation correlates with cardiomyocyte cell death and impaired cardiac function [22]. In adult cardiomyopathy, cardiac-specific MDM4 deletion triggers p53-dependent apoptosis, leading to dilated cardiomyopathy with sex-dimorphic progression. Notably, MDM4 loss exacerbates pressure overload-induced remodeling, while its preservation protects against stress-induced heart failure, underscoring its essential role in cardiomyocyte survival [23, 37]. Our results demonstrate that Dox can decrease MDM4 expression in H9C2 cardiomyocytes. Mechanistically, our findings identify USP8 as a novel stabilizer of MDM4 via deubiquitination. Moreover, inhibition of MDM4 abolishes the protective effects of USP8 overexpression, confirming MDM4 as a downstream mediator in mitigating Dox-induced cell death and ferroptosis. These findings underscore MDM4 as a pivotal effector in USP8-mediated cardioprotection.

However, several limitations warrant consideration. First, this study exclusively utilized the H9C2 rat cardiomyocyte line, which may not fully recapitulate the complexity of human cardiac pathophysiology or in vivo stress responses. Second, while our study identified MDM4 stabilization as a key mechanism, the downstream effectors linking MDM4 to ferroptosis and cell death regulation, particularly its interplay with p53 or other ubiquitination-related pathways, remain incompletely characterized. Third, these experiments lack the complexity of primary cardiomyocytes or in vivo models. Future studies should prioritize three directions. First, validating these findings in in vivo models would strengthen clinical relevance. Second, identifying upstream regulators of USP8 and downstream MDM4 effectors will elucidate broader regulatory networks. Finally, developing tissue-specific USP8 activators or MDM4 stabilizers could pave the way for novel interventions against AHF. Additionally, oeUSP8 group shows a value of approximately 3 ru with high significance (2 asterisks) in Fig. 2A, while it is around 2.0 ru with highly significant difference (3 asterisks) in Supplementary Fig. 2. The apparent discrepancy in statistical significance stems from different statistical methods (Student’s *t*-test in Fig. 2A and one-way ANOVA in Supplementary Fig. 2), data variability, and distinct normalization approaches.

Collectively, our study demonstrates that USP8 overexpression protects H9C2 cardiomyocytes from Dox-induced ferroptosis through its deubiquitination-dependent stabilization of MDM4.

## Supplementary Information

Below is the link to the electronic supplementary material.

Supplementary Figure 1: Schematic of the interaction between USP8 and MDM4 using the UbiBrowser^2.0^ bioinformatics tool (http://ubibrowser.bio-it.cn/ubibrowser_v3/home/index)

Supplementary Figure 2: USP8 expression in H9C2 cardiomyocytes transfected with or without oeNC or oeUSP8. ***P < 0.001

Supplementary Figure 3: USP8 and MDM4 levels in treated H9C2 cardiomyocytes. ***P < 0.001

Supplementary Figure 4: Effects of MDM4 overexpression on Dox-triggered cell death and ferroptosis in H9C2 cells. H9C2 cells after 24 h transfection with or without oeNC or oeMDM4 were stimulated with or without 1 µM Dox for 24 h. The influences on MDM4 expression (**A**), cell viability (**B**), LDH release (**C**), cell death (**D**), Fe^2+^ levels (**E**), lipid ROS production (**F**), MDA and GSH expression (**G** and **H**), and GPX4 and SLC7A11 levels (**I**) were evaluated. *P < 0.05, **P < 0.01, ***P < 0.001

Supplementary Material 5

## Data Availability

No datasets were generated or analysed during the current study.
