# Supplementary figures and images for: USP8 protects rat-derived H9C2 cardiomyocytes from doxorubicin-triggered ferroptosis and cell death through deubiquitination-mediated stabilization of MDM4
Source: Hereditas. 2025 Aug 14;162:158. doi: 10.1186/s41065-025-00527-z (PMC12351941; doi:10.1186/s41065-025-00527-z)

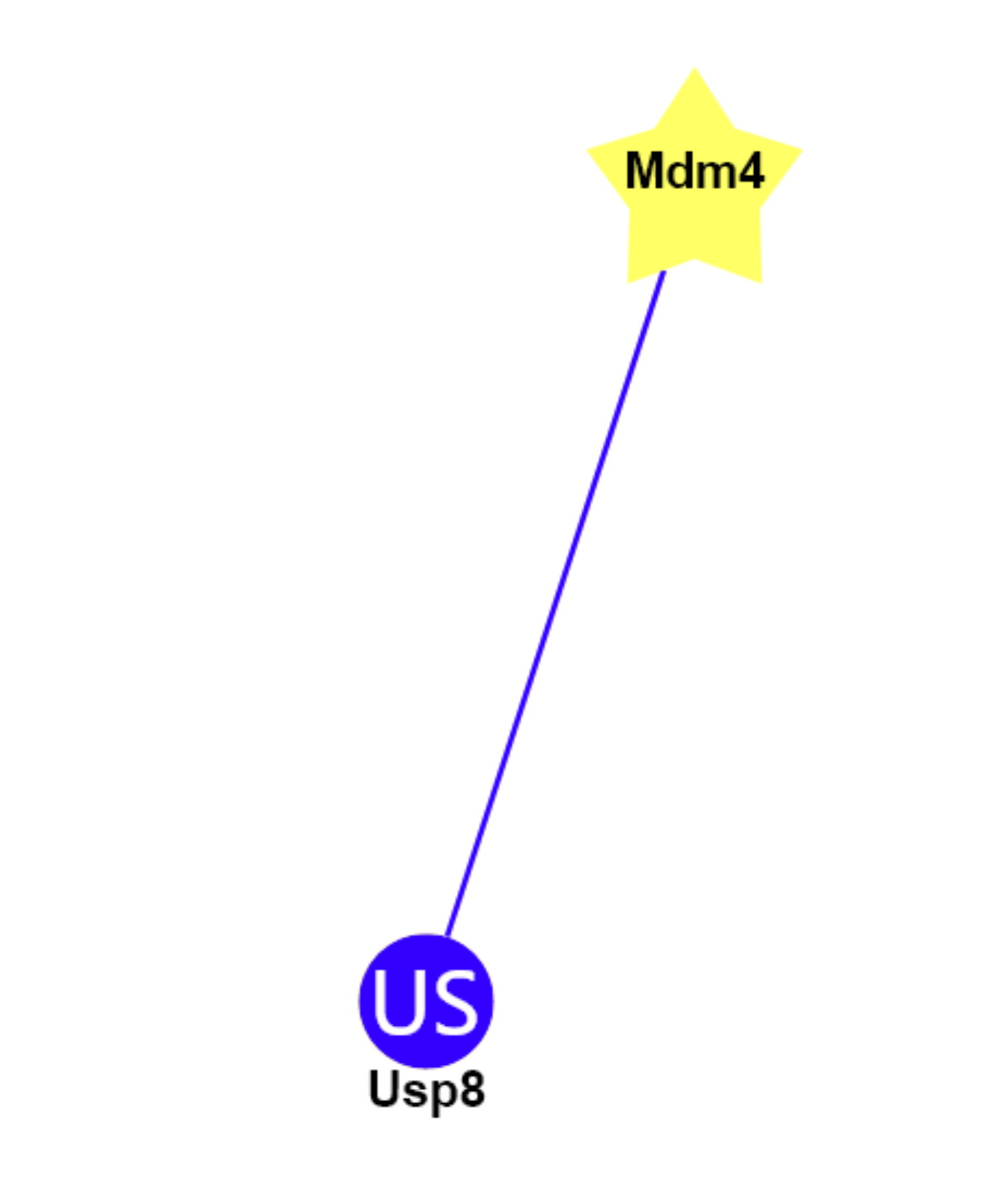

Supplement: Supplementary file 1 — Supplementary Figure 1: Schematic of the interaction between USP8 and MDM4 using the UbiBrowser2.0 bioinformatics tool (http://ubibrowser.bio-it.cn/ubibrowser_v3/home/index) [file 41065_2025_527_MOESM1_ESM.tif]

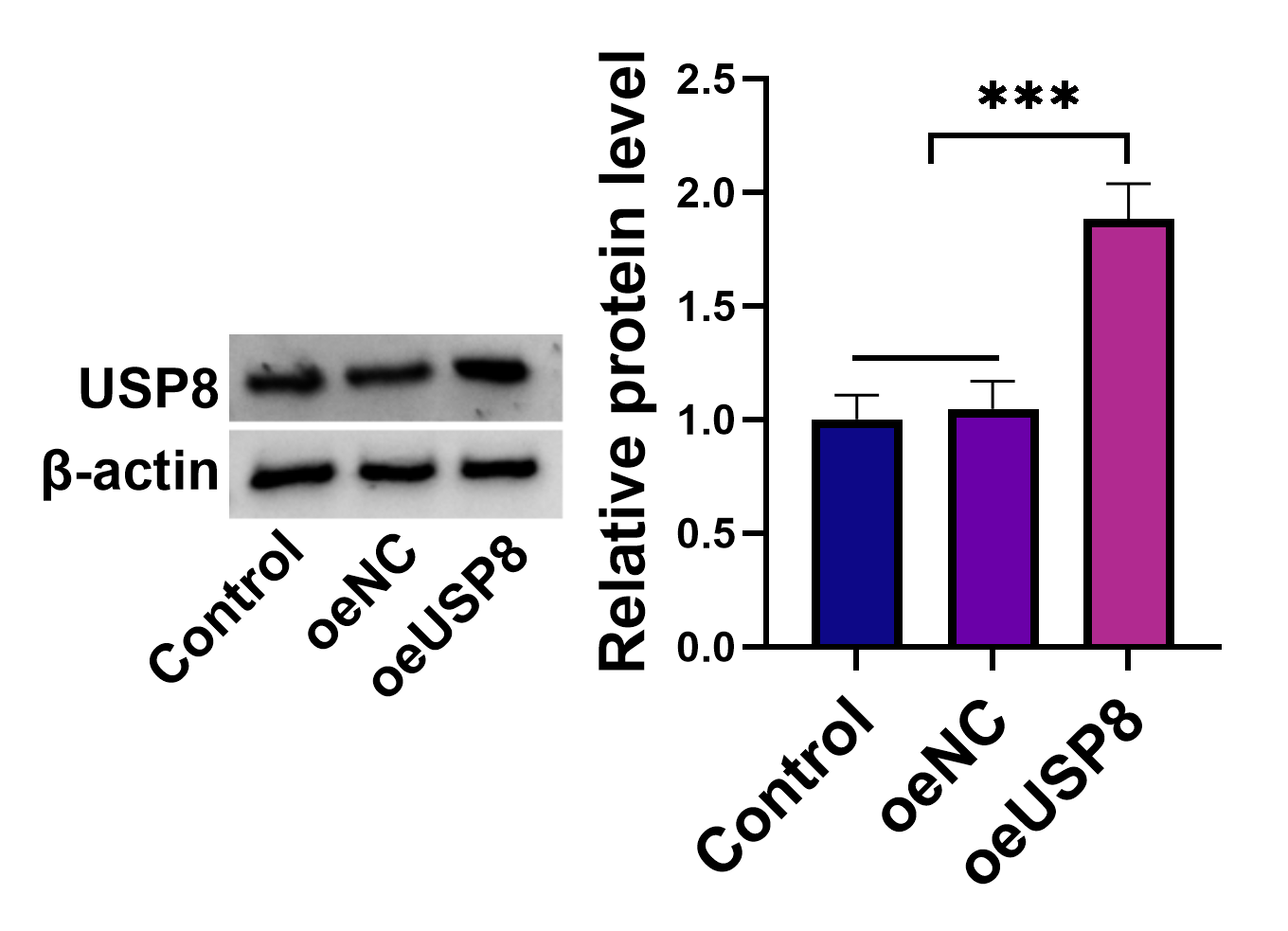

Supplement: Supplementary file 2 — Supplementary Figure 2: USP8 expression in H9C2 cardiomyocytes transfected with or without oeNC or oeUSP8. ***P < 0.001 [file 41065_2025_527_MOESM2_ESM.tif]

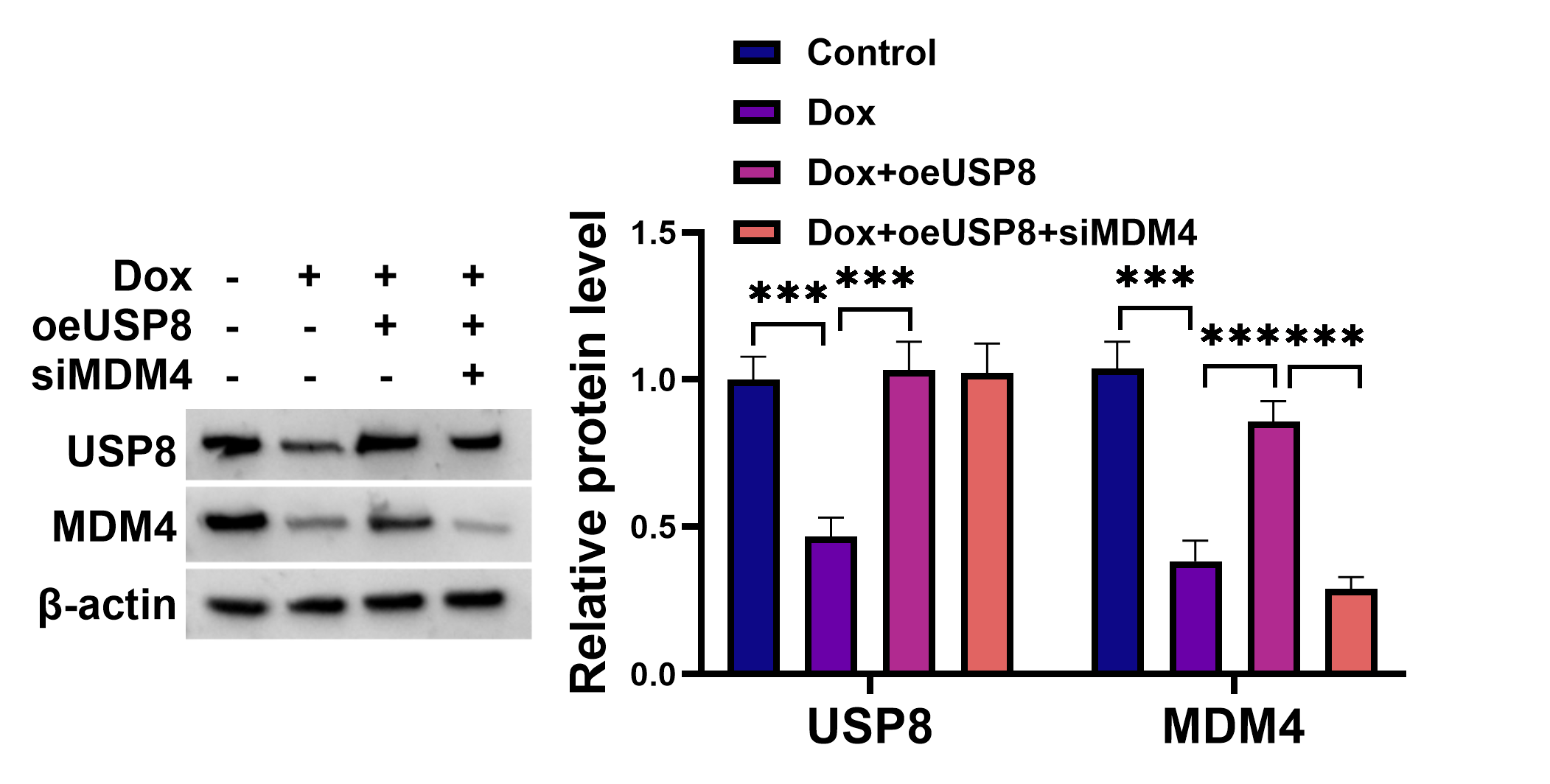

Supplement: Supplementary file 3 — Supplementary Figure 3: USP8 and MDM4 levels in treated H9C2 cardiomyocytes. ***P < 0.001 [file 41065_2025_527_MOESM3_ESM.tif]

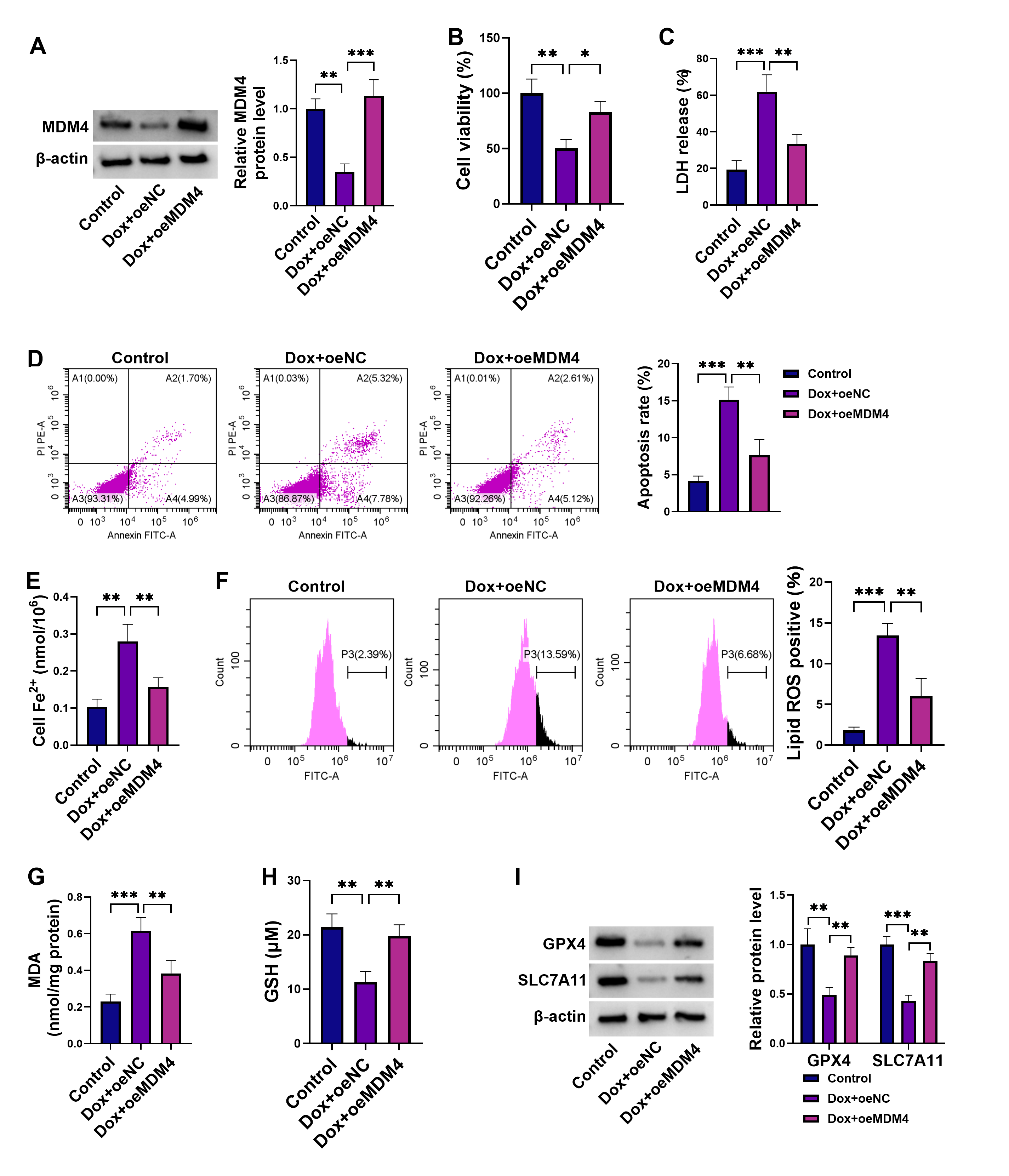

Supplement: Supplementary file 4 — Supplementary Figure 4: Effects of MDM4 overexpression on Dox-triggered cell death and ferroptosis in H9C2 cells. H9C2 cells after 24 h transfection with or without oeNC or oeMDM4 were stimulated with or without 1 µM Dox for 24 h. The influences on MDM4 expression (A), cell viability (B), LDH release (C), cell death (D), Fe2+ levels (E), lipid ROS production (F), MDA and GSH expression (G and H), and GPX4 and SLC7A11 levels (I) were evaluated. *P < 0.05, **P < 0.01, ***P < 0.001 [file 41065_2025_527_MOESM4_ESM.tif]
