# Supplementary material for: USP8 protects rat-derived H9C2 cardiomyocytes from doxorubicin-triggered ferroptosis and cell death through deubiquitination-mediated stabilization of MDM4
Source: Hereditas. 2025 Aug 14;162:158. doi: 10.1186/s41065-025-00527-z (PMC12351941; doi:10.1186/s41065-025-00527-z)

Fig.1C

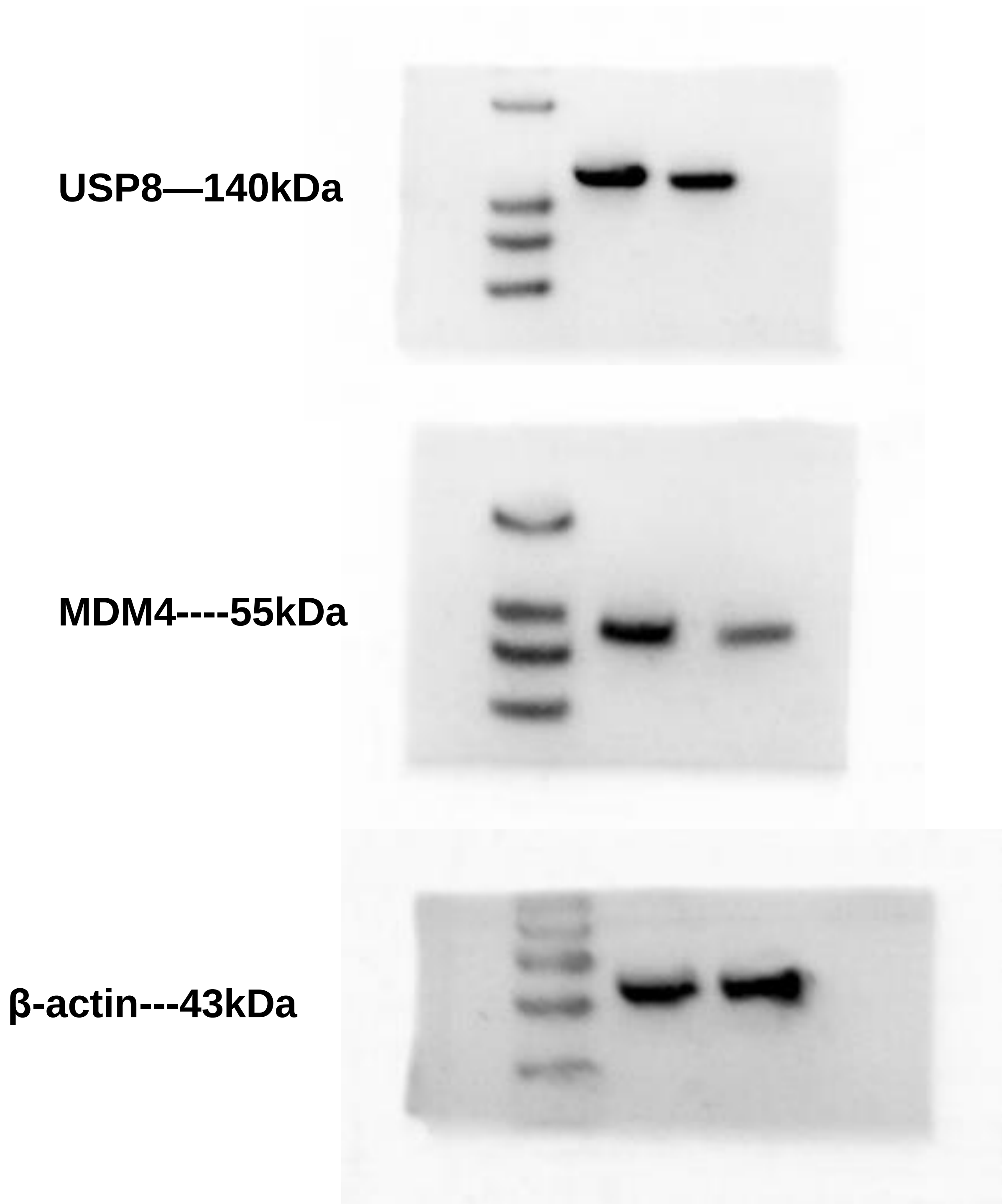

Fig.2A

USP8—140kDa

β-actin---43kDa

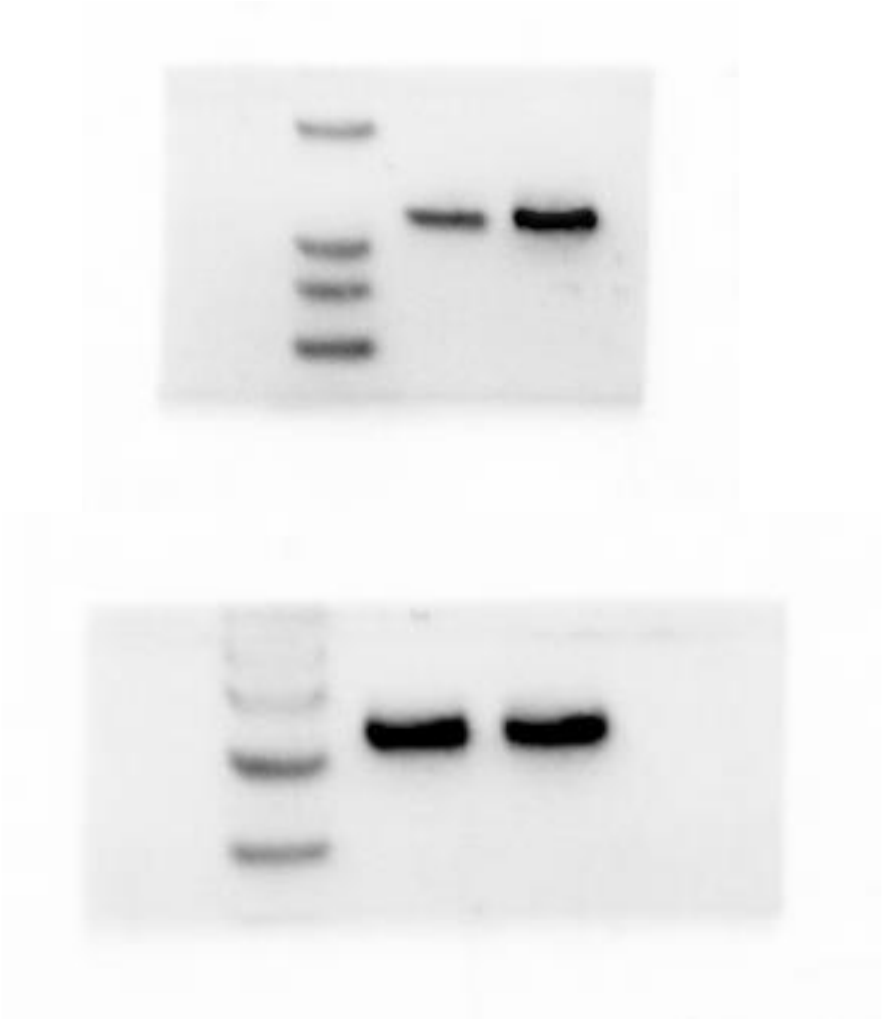

Fig.3E

GPX4---17kDa

SLC7A11---35kDa

β-actin---43kDa

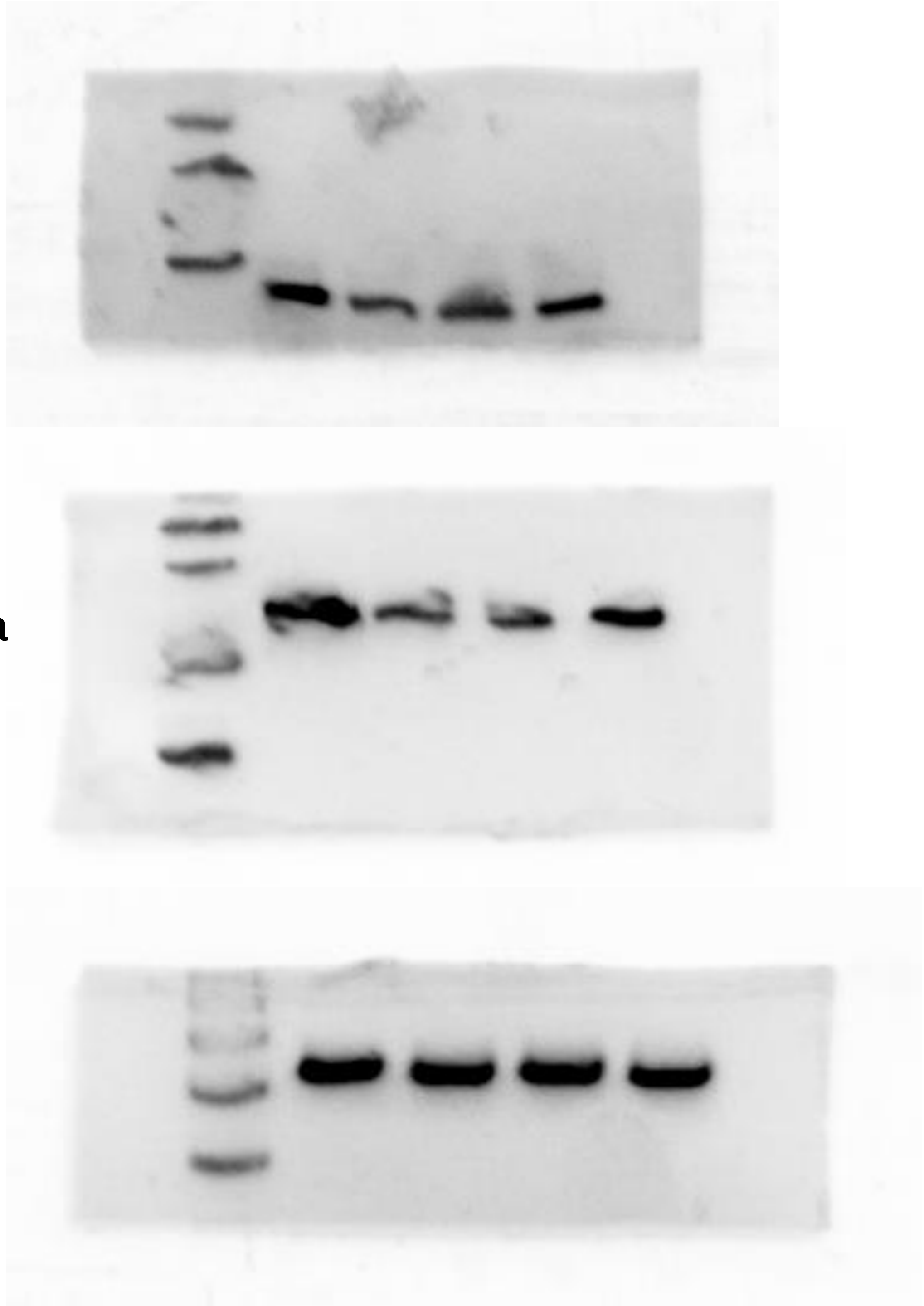

Fig.4A

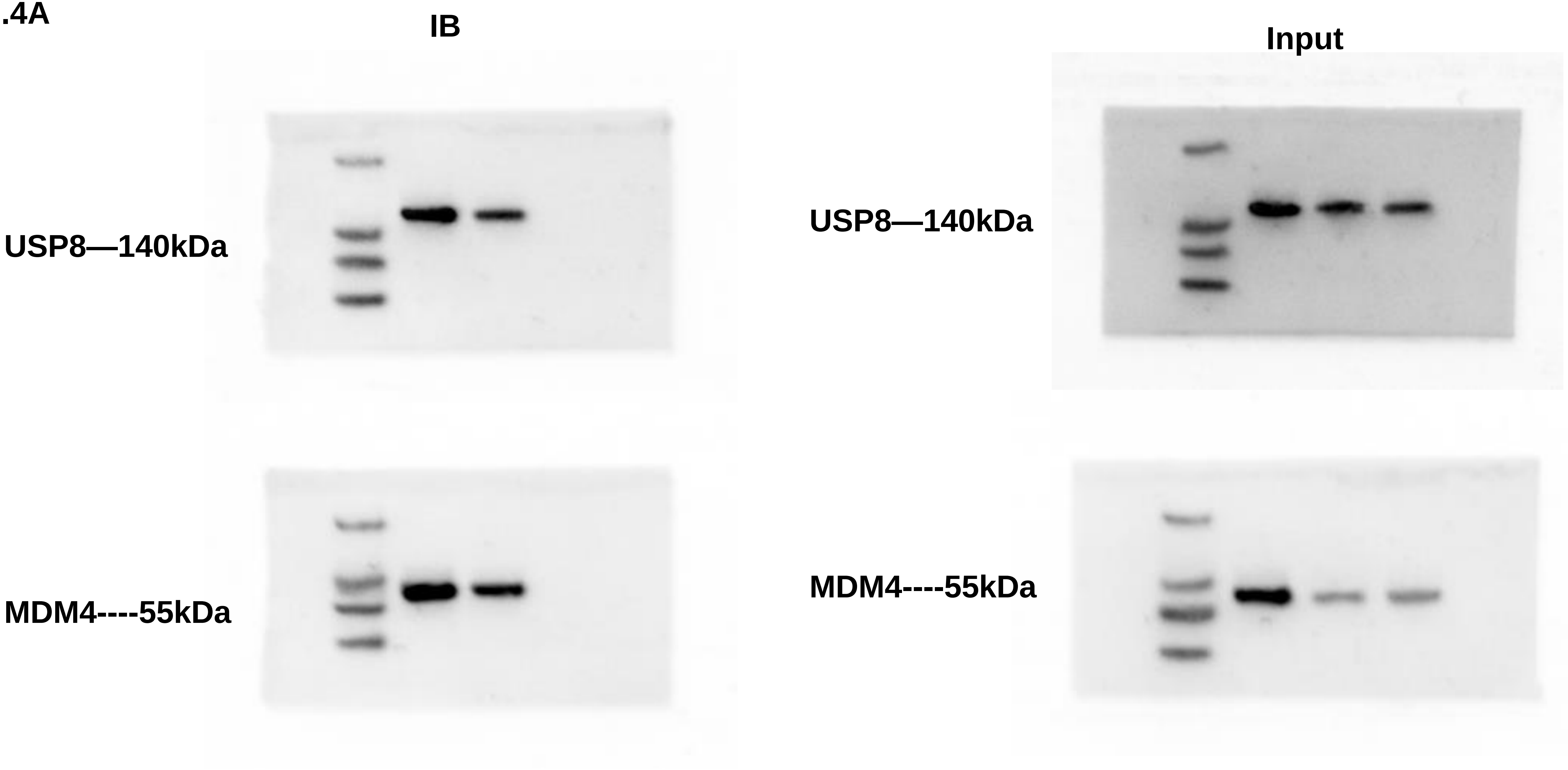

Fig.4B

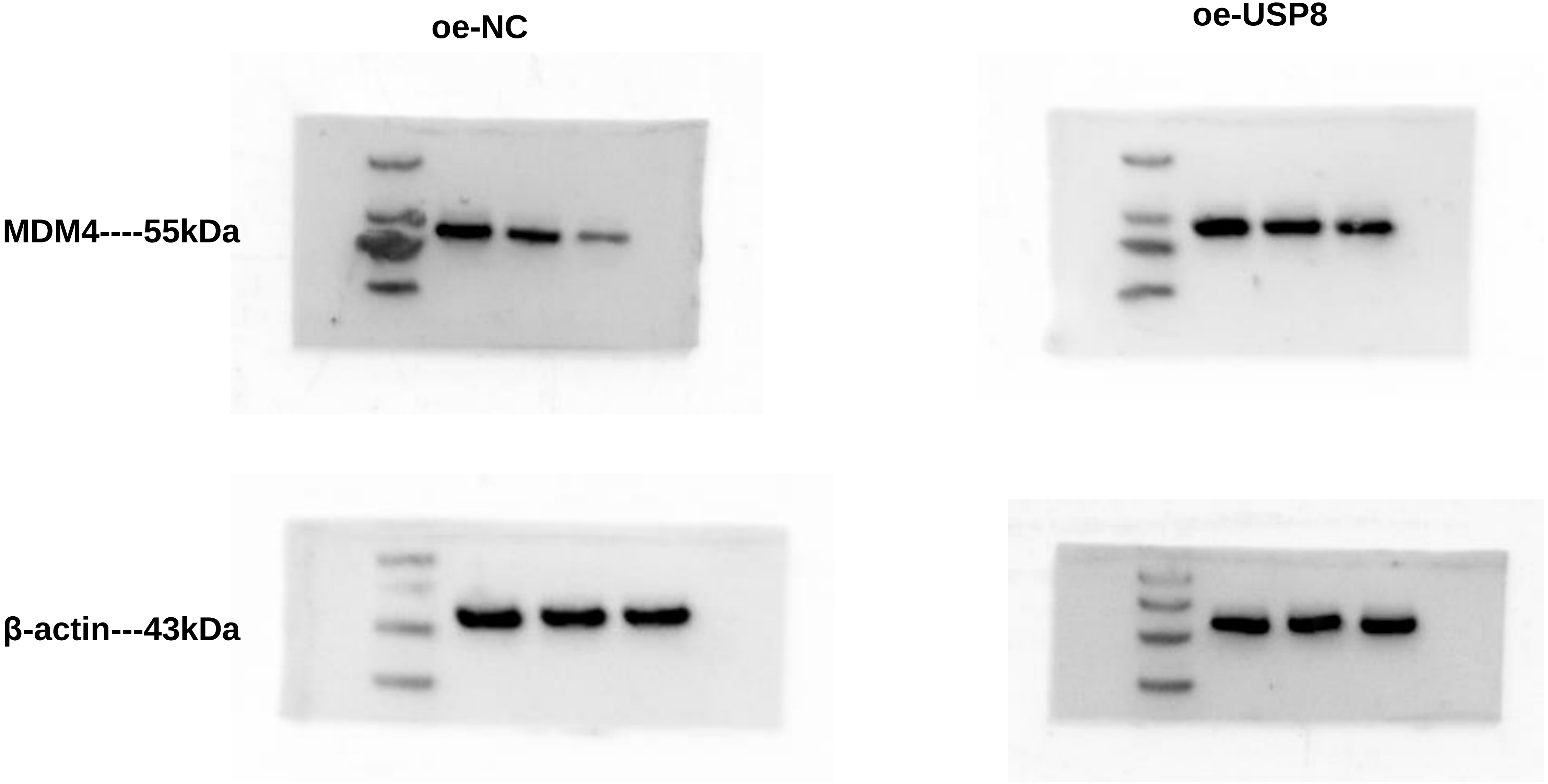

Fig.4C

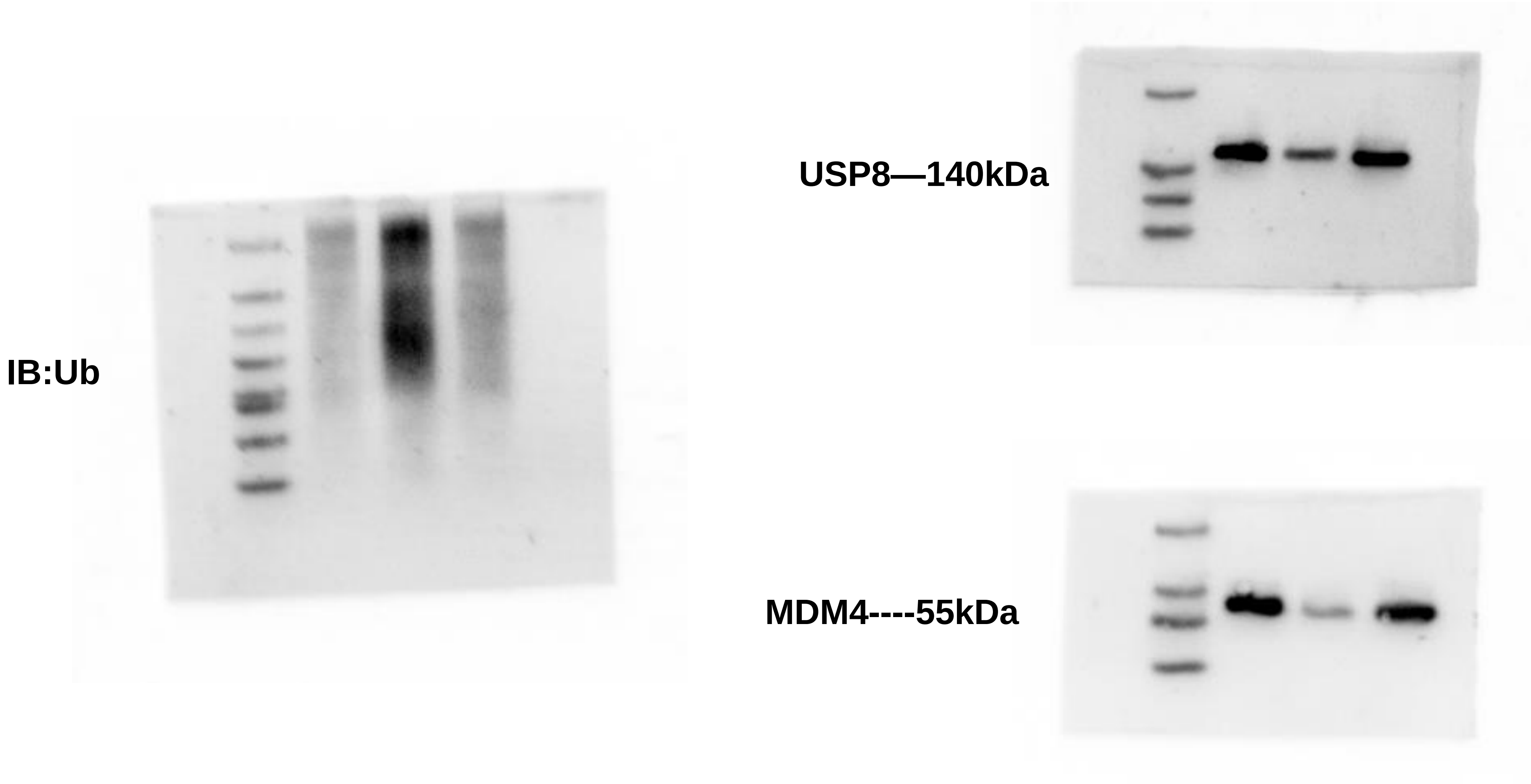

Fig.4D

MDM4----55kDa

β-actin---43kDa

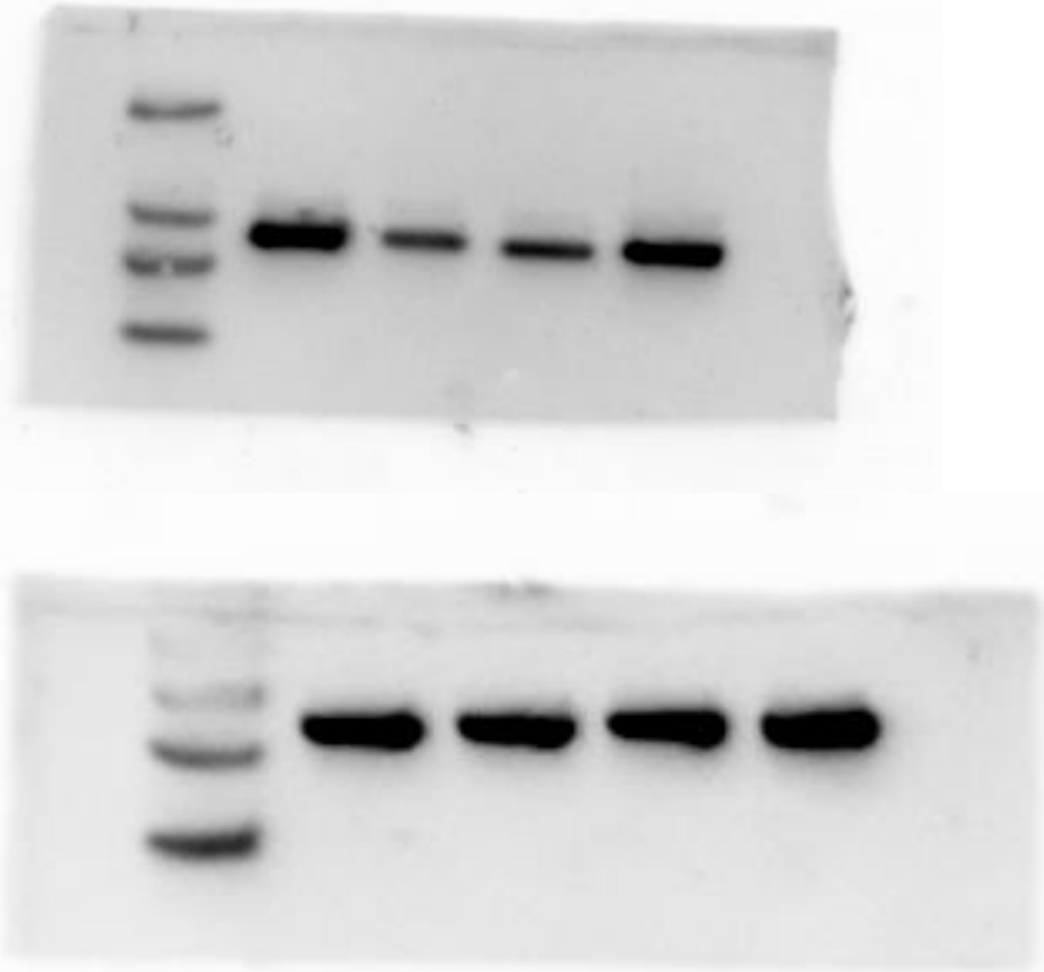

Fig.5A

MDM4----55kDa

β-actin---43kDa

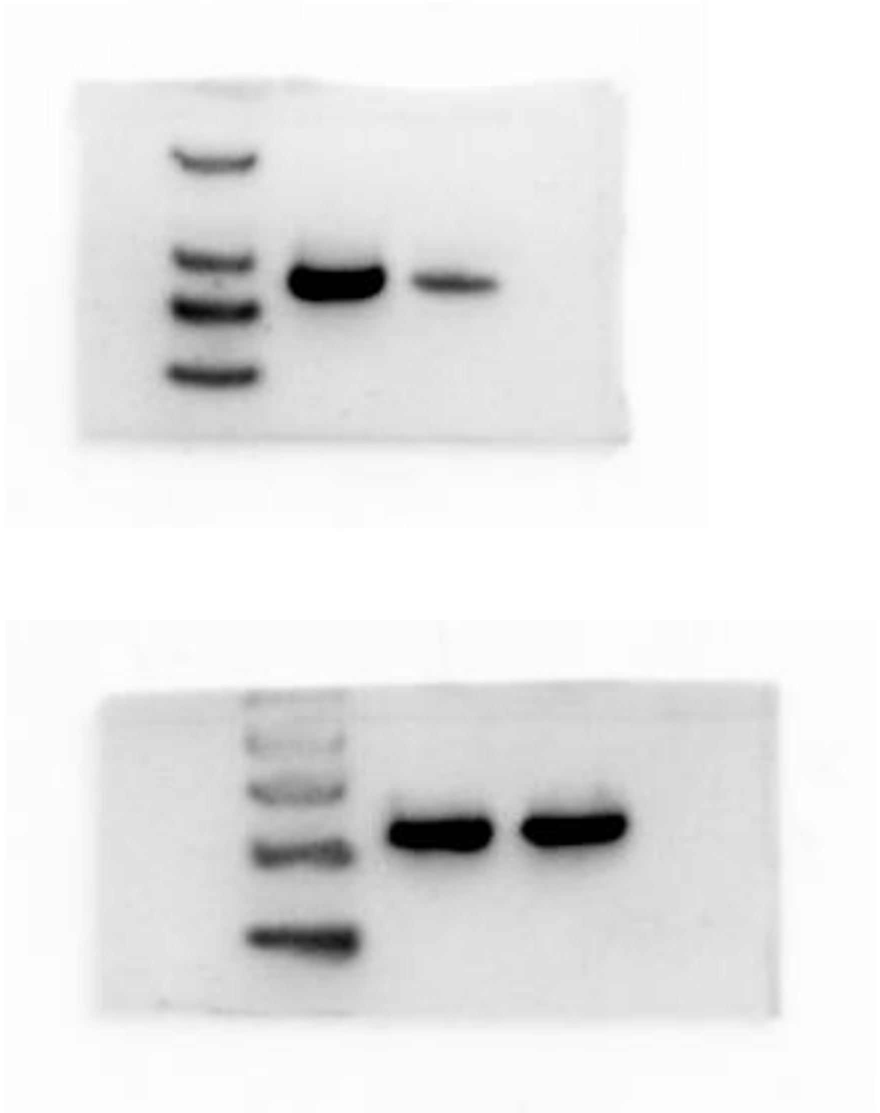

Fig.5I

GPX4---17kDa

SLC7A11---35kDa

β-actin---43kDa

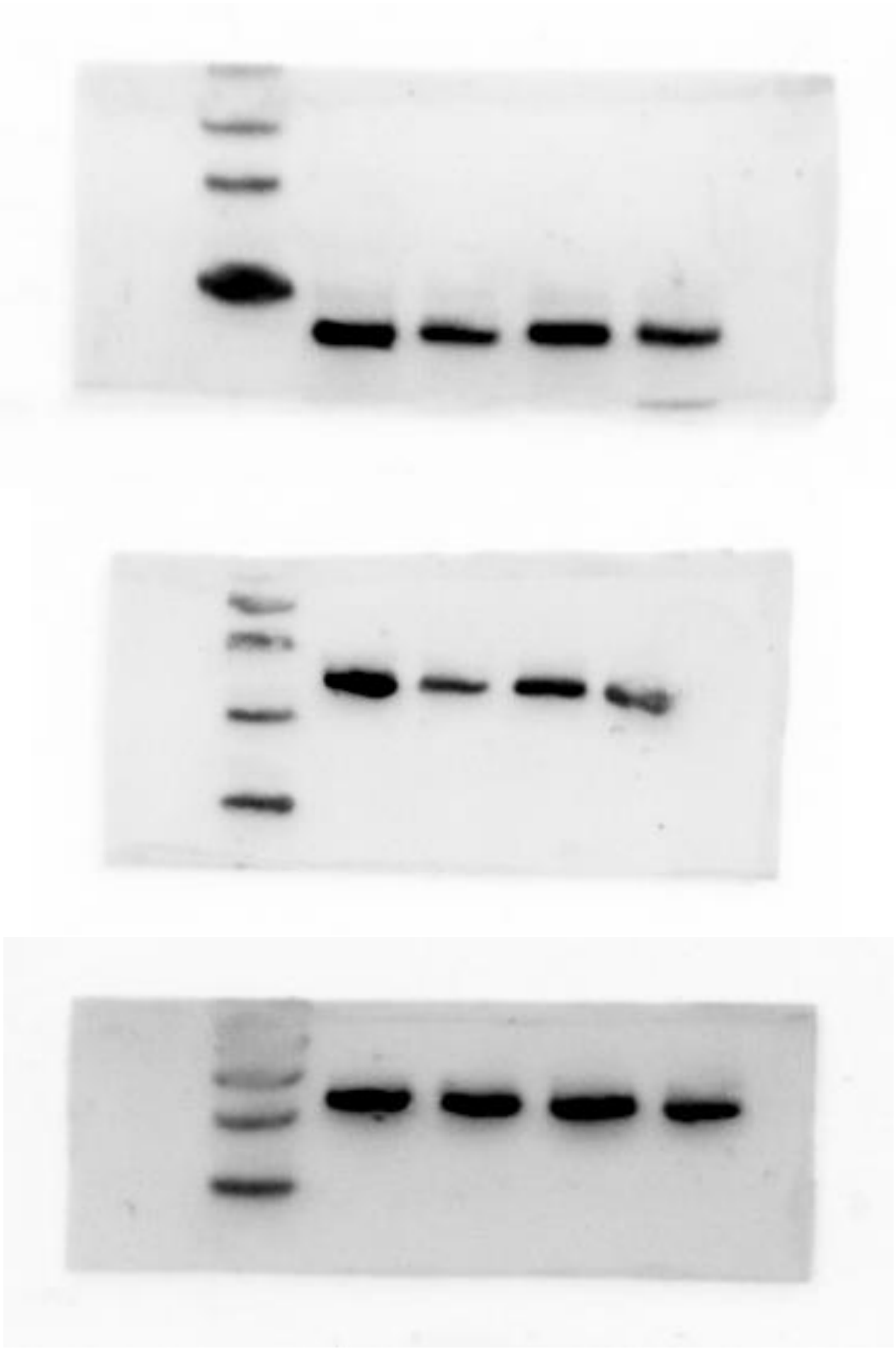

marker

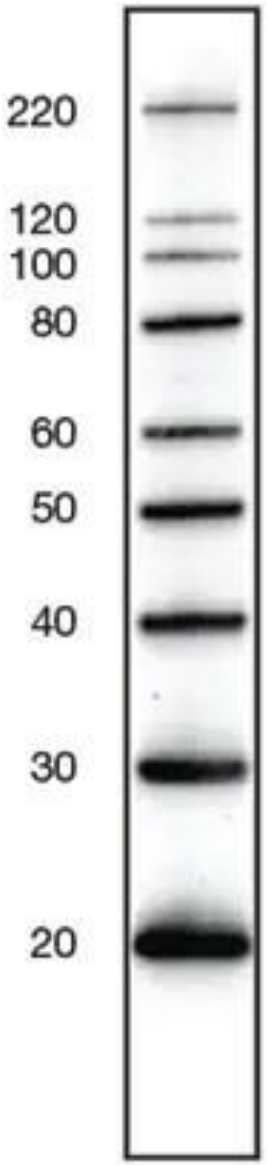

Supplement: Supplementary file 5 — Supplementary Material 5 [file 41065_2025_527_MOESM5_ESM.pdf]
